# Morel (*Morchella* spp.) intake alters gut microbial community and short-chain fatty acid profiles in mice

**DOI:** 10.3389/fnut.2023.1237237

**Published:** 2023-09-22

**Authors:** Longying Pei, Wei Liu, Luping Liu, Xiaoyu Wang, Luxi Jiang, Zhaohui Chen, Qiquan Wang, Peng Wang, Heng Xu

**Affiliations:** ^1^College of Food Science and Engineering, Xinjiang Institute of Technology, Aksu, Xinjiang, China; ^2^College of Food Science and Engineering, Tarim University, Alar, Xinjiang, China; ^3^Zhiran Biotechnology Co., Ltd, Tianjin, China

**Keywords:** morel, gut microbiota, short-chain fatty acids, mice, obesity reduction

## Abstract

Morels (*Morchella* spp.) are highly nutritious and consumed as both edible mushrooms and traditional Chinese medicine. This study aimed to investigate the effects of dietary supplementation with morel mushrooms on the gut bacterial microbiota and short-chain fatty acids (SCFAs) profiles in healthy mice. Healthy mice were randomly assigned to five groups: a control group (0% morel) and four intervention groups supplemented with different levels of morel mushrooms (5% for M5, 10% for M10, 15% for M15, and 20% for M20) over a period of 4 weeks. Fecal samples were collected at the end of the experiment to characterize the microbiota and assess the SCFAs levels. The morel intervention significantly altered the bacterial community composition, increasing *Bacteroides*, *Lachnospiraceae NK4A136 group* and *Parabacteroides*, while decreasing *Staphylococcus* and the Firmicutes to Bacteroidetes ratio (F/B ratio). Moreover, increased morel intake was associated with weight loss. All SCFAs content was upregulated in the morel-intervention groups. Potential SCFAs-producing taxa identified by regression analysis were distributed in the families *Muribaculaceae*, *Lachnospiraceae*, and in the genera *Jeotgalicoccus, Gemella, Odoribacter*, *Tyzzerella 3* and *Ruminococcaceae UCG-014*. The functional categories involved with SCFAs-production or weight loss may contain enzymes such as beta-glucosidase (K05349), beta-galactosidase (K01190), and hexosaminidase (K12373) after morel intervention. The exploration of the impact of morel mushrooms on gut microbiota and metabolites contributes to the development of prebiotics for improving health and reducing obesity.

## 1. Introduction

Edible mushrooms are highly valued by consumers because of their distinctive flavor and nutritional benefits. They are excellent sources of protein, carbohydrates, vitamins, and trace elements, making them an essential part of a healthy diet (1). Morels which belong to the *Morchella* species within the Pezizales order of the Ascomycota family, are named after their honeycomb-shaped cap, which resembles the stomach of a sheep. They are mainly grown in southwest, northwest and central China, and are considered a rare delicacy with medical properties. Morels are rich in essential amino acids, dietary fibers, vitamins, minerals, and other nutrients (2). Morels have long been used as traditional Chinese medicine for the prevention and treatment of diseases due to their bioactive and beneficial substances such as polysaccharides, phenols, and terpenoids. Morels are known to have a regulatory effect on the stomach and are believed to have a tonifying effect on the kidney (3). As result, their demand has grown rapidly in recent years due to the discovery of numerous healthy benefits (4).

Accumulating evidence shows that microbes and metabolites in the human gut have a close relationship with health (5). Gut microbiota plays a vital role in host metabolism, energy expenditure, nutrient absorption, immune system development and regulation, and maintenance of good health (6). Dysbiosis of gut microbiota has been linked to a range of health problems (7, 8). The composition of the gut microbiota is influenced by several environmental factors, such as drug overdose, mental stress and diets and lifestyle habits, particularly diets (9). Therefore, targeting the gut microbiota through specific dietary or prebiotic interventions has been recognized as an effective preventive approach for addressing several health concerns (10). For example, studies have demonstrated that the consumption of dietary fiber and prebiotics can increase the abundance of beneficial bacteria in the gut, such as *Bifidobacterium* and *Lactobacillus*, while decreasing the harmful bacteria like *Clostridium difficile* and Enterobacteriaceae (11). In addition, dietary interventions such as the Mediterranean diet have been found to promote a more diverse gut microbiota and may be associated with a reduced risk of several chronic diseases (12). Furthermore, research has suggested that prebiotics, such as inulin and oligofructose, may have beneficial effects on gut microbial composition and function, resulting in health benefits for the host (13).

Edible mushrooms contain high levels of bioactive compounds including hemicellulose, chitin, α and β-glucans, mannans, galactans, xylans, and polyphenols, which promote the growth of beneficial microorganisms and are a potential source of prebiotics (14), edible mushrooms or extracts thereof are now widely used in dietary supplements and functional foods. Highlighting the impact of these mushrooms on gut health, a study conducted in an *in vitro* fermentation model revealed notable variations in SCFAs levels and pH among six different edible mushrooms, this investigation demonstrated substantial shifts in the composition and diversity of gut microbiota. Specifically, *Auricularia auricular* increased the abundance of Bifidobacteriales and Bacteroidales while reducing Fusobacteriales. Similarly, *Agaricus bispours* contributed to an elevated abundance of *Lactobacillus*. These findings underscore the potential of common edible mushrooms to enhance gut health through the modulation of gut microbiota and SCFAs production (15). Another study reported that consumption of white button mushrooms increased the abundance of Lachnospiraceae and Ruminococcacea, as well as changes in carbohydrate metabolism and secondary metabolite biosynthesis in pigs, Lachnospiraceae has the potential to act as a probiotic, fermenting carbohydrates to produce acetic and butyric acids, which serve as a source of energy for the host (16). Likewise, mice fed white button mushrooms experienced notable alterations in the gut microbiota, manifesting as enhanced diversity and an expansion of *Bacteroidetes* members (17). Considerable research has investigated the probiotic effects of edible mushrooms and extracts, particularly their polysaccharides, in improving the gut microbiota. Studies suggest that morel polysaccharides are effective in improving the structure of the gut microbiota, thereby reducing body weight and modulating the immune system through flora regulation (18). However, the impact of morels on host gut microbiota and metabolites still remains unclear.

The objective of this study is to examine the impact of various levels of morel intervention on the diversity and composition of gut microbiota. Using healthy mice as animal models, we explored the effects of morel intake on bacterial community composition, microbiota function, and SCFAs content, and analyzed their associations. Our findings suggest that morel intervention can effectively modulate intestinal microbiota, leading to weight control and reduction. Overall, this work sheds light on the potential benefits of morels in promoting gut health and reducing obesity.

## 2. Materials and methods

### 2.1. Animal experiment

The Morels were collected from Xinjiang Hanyi Ecological Agriculture Technology Development Co., Ltd. and transported to the laboratory. The samples were dried at 42°C, then powered and sieved to ensure the removal of large particles. The diet for mice was prepared by adding a proportion of morel powder to the standard AIN-93G diet. Animal experiments were conducted with the approval of the Institutional Animal Care and Use Committee (IACUC) in China [IACUC-2022-0610]. Specific pathogen-free male C57BL/6 J mice (8 weeks old, 18–22 g weight) were housed for a week with free access to a standard diet and water in a 12 h light/dark environment. The mice were accommodated in 10 cages with three mice in each cage. After a 1 week acclimation period, these mice were randomly assigned to five groups (*n* = 6 per group). The control group was fed with a standard AIN-93G diet. The group of M5, M10, M15, and M20 was fed with a standard diet added with 5, 10, 15, and 20% morel powder, respectively. Body weight and food intake were recorded each week. After 4 weeks of feeding, these mice were killed by CO_2_ and cervical dislocation. Then fecal samples were collected and stored at −80°C for further analysis.

### 2.2. Profiling of short chain fatty acids

The fecal SCFAs (acetate, propionate, valerate, isovalerate, butyrate and isobutyrate) were determined by gas chromatography–mass spectrometry (GC–MS) system using Agilent 7890B/7000D (Agilent Technologies, Santa Clara, CA, United States). The sample preparation and detection conditions were referred to previous study (19).

### 2.3. Amplicon sequencing

Total genomic DNA from all the feces was extracted using the hexadecyl trimethyl ammonium bromide (CTAB) method (20). DNA concentration and purity were monitored using 1% agarose gel. DNA was diluted to a final concentration of 1 ng/μL using sterile distilled water. The 16S rRNA gene V3–V4 region was PCR-amplified using the following specific primers: 314F 5′- CCTAYGGGRBGCASCAG-3′ and 806R 5’-GGACTACHVGGGTWTCTAAT-3′ with barcodes. All PCR reactions were carried out in 30 μL reactions with 15 μL of Phusion^®^ High-Fidelity PCR Master Mix (New England Biolabs), 0.2 μM of forward and reverse primers, and approximately 10 ng template DNA. Thermal cycling consisted of initial denaturation at 98°C for 1 min, followed by 30 cycles of denaturation at 98°C for 10 s, annealing at 50°C for 30 s, and elongation at 72°C for 30 s, and finished by a final extension at 72°C for 5 min. Amplicons were purified with Qiagen Gel Extraction Kit (Qiagen, Germany). Sequencing libraries were generated using TruSeq^®^ DNA PCR-Free Sample Preparation Kit (Illumina, United States) following manufacturer’s recommendations and index codes were added. The library quality was assessed on the Qubit@ 2.0 Fluorometer (Thermo Scientific) and Agilent Bioanalyzer 2,100 system. At last, the library was sequenced on an Illumina NovaSeq platform and 250 bp paired-end reads (PE 250) were generated. Raw sequencing data were deposited at NCBI with BioProject accession number PRJNA904704.

### 2.4. Sequencing data processing

All raw paired-end sequences were imported to QIIME2 pipeline (version 2022.2) (21). The barcodes were removed using Cutadapt. The paired-end sequences were merged using Vsearch. The reads with low quality were removed by Trimmomaitc. Then clean reads were used to generate amplicon sequence variant (ASV) and ASV table by the DADA2 plugin with default parameters (22). Taxonomic annotation was performed using Silva132 (16S_V34) database (23). The ASVs belonging to chloroplasts and mitochondria were removed. We use PICRUSt2 software^1^ to predict the functional abundance of microbiota (24).

### 2.5. Statistical analysis

Alpha diversity was calculated via “qiime diversity alpha” command by QIIME2. The non-parametric Wilcoxon rank sum test was used to analyze the signification of the difference of α diversity between the two groups. Principle coordinates analysis (PCoA) analysis and non-metric multidimensional scaling (NMDS) was performed using R in-house scripts and visualized by “qiime emperor plot.” Permutational analysis of variance (PERMANOVA) was applied to test group differences based on a distance matrix using the vegan package. LEfSe (Linear discriminant analysis Effect Size) was used to test the difference in taxa abundance and functional abundance (25). The lm function was used for linear regression analysis between the top 10 species and the levels of morel intake. R-squared values were calculated, and *F*-tests were used to determine the corresponding *p*-values. We used MaAsLin2 (26), a modified general linear model for feature-wise multivariate modeling, to identify differentially abundant taxa or KEGG pathways associated with SCFAs.

## 3. Results

### 3.1. Morel intervention altered mice gut microbial diversity

**Figure 1:**
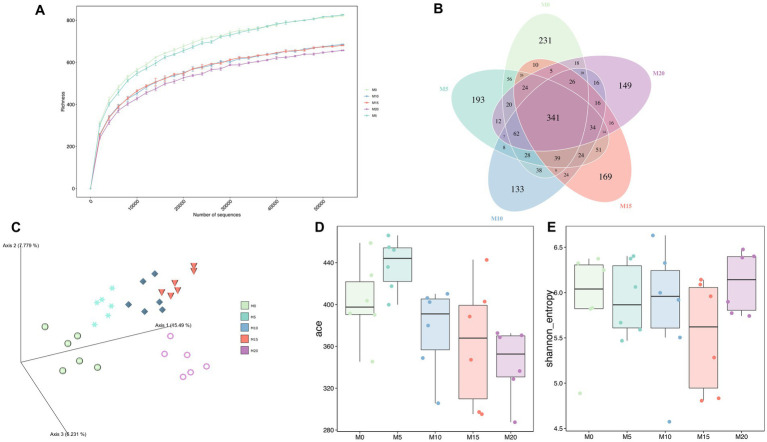
The bacterial microbiota diversity following morel intervention. **(A)** Rarefaction curve. **(B)** Venn diagram between control and morel-intervention groups. **(C)** PCoA plot based on aitchison distance. **(D)** ACE richness index. **(E)** Shannon diversity index.

To determine the effect of morel intervention on mice gut microbiota, we performed a V3–V4 region amplicon sequencing analysis. We obtained a total of 2,551,011 raw reads, of which 2,289,357 clean reads were retained after applying a quality filter, resulting in the identification of 1,852 amplicon sequencing variants (ASVs). With the increase of sequencing depth, the species richness tended to be flat (Figure 1A), suggesting the sequencing depth was adequate to represent the microbial diversity among different groups.

Venn diagrams showed that 341 ASVs (18.41%) were shared across various groups (Figure 1B). Additionally, we identified over 100 ASVs unique in each group, with M0, M5, M10, M15, and M20 containing 231, 193, 133, 169, and 149 ASVs, respectively. These unique ASVs accounted for 47.25% of the total ASVs. Moreover, PCoA analysis indicated a significant separation between samples from different groups (Adonis *R*^2^ = 0.471, *p* = 0.001) (Figure 1C), suggesting that morel intervention altered the composition of mice gut microbiota. Meanwhile, these groups exhibited an arcuate trajectory in the PCoA plot as the increasing morel content addition.

α-diversity indexes were used to evaluate microbial diversity across different groups (Figures 1D,E; Supplementary Table S1). Compared to the control group M0, ACE index did not display significant changes in M5, M10, and M15 (*p* > 0.05), but it significantly decreased in M20 (*p* = 0.011), indicating that high-dose morel intervention led to a reduction in microbial richness. Although no significant difference was noted among groups in Shannon index, there was still a decreasing trend, except for M20, suggesting that morel intervention did not significantly affect the microbiota evenness (*p* > 0.05).

### 3.2. Morel intervention altered the bacterial community composition

All ASVs were assigned to 11 phyla, 18 classes, 34 orders, 66 families, 171 genera, and 100 species. The top 10 bacterial taxa at phylum and species levels were identified in both control and morel-intervened groups, albeit with different relative abundances. At the phylum level, Bacteroidetes, Firmicutes, and Proteobacteria were the dominant microbial taxa (relative abundance >1%) (Supplementary Figure S1A). Morel intervention increased the relative abundance of Bacteroidetes and decreased the relative abundance of Firmicutes, Proteobacteria and Actinobacteria. The F/B ratio was decreased after morel intervention and negatively correlated with the proportion of morel intake (Supplementary Figure S1B). Moreover, we found that host weight had a similar trend with the change in F/B ratio (Supplementary Figure S1C), which was in line with a previous study (27, 28). As morel intake increased, there was a significant rise in the mice’s body weight (Supplementary Figure S1D). However, an increase in morel consumption was evidently associated with a decrease in body weight (Supplementary Figure S1E).

**Figure 2:**
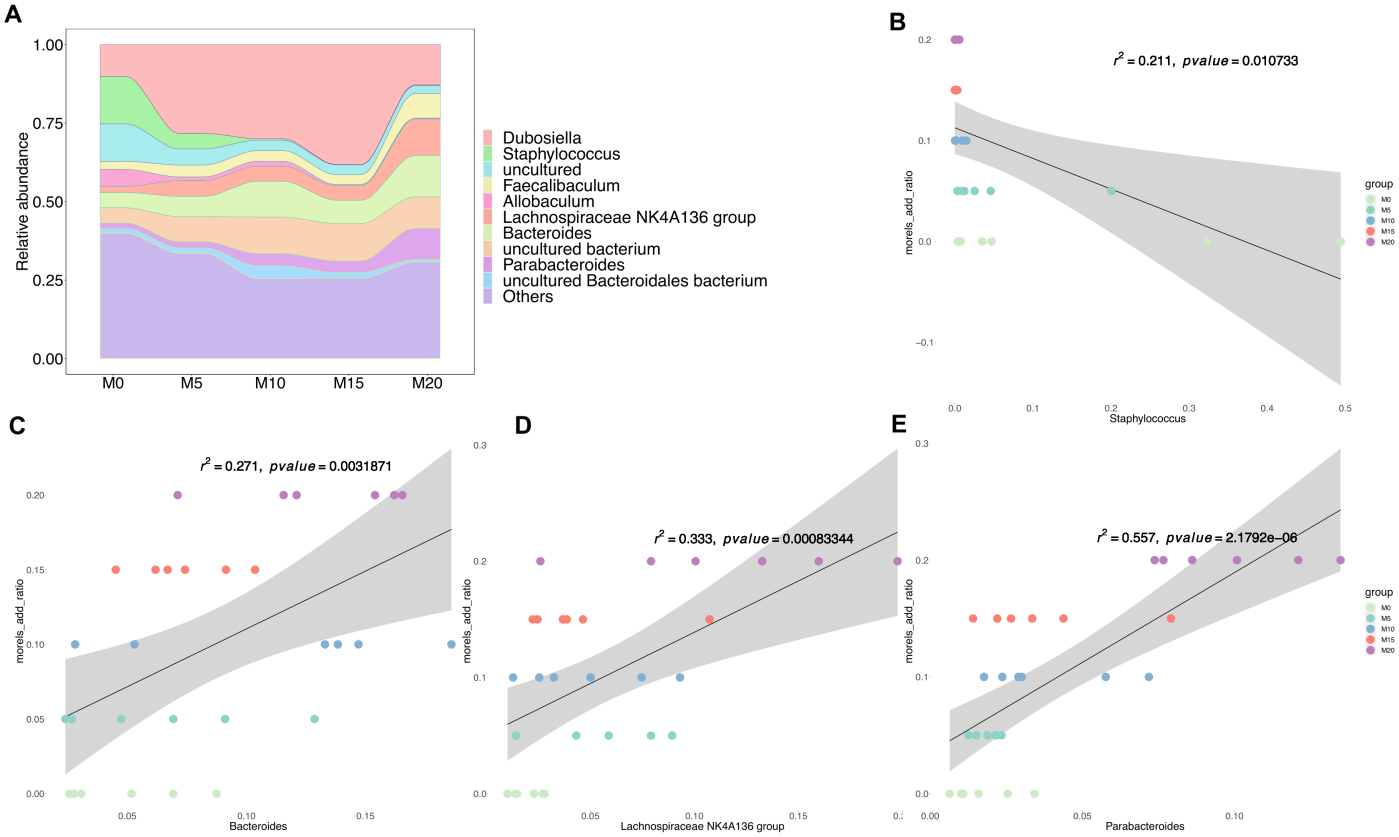
The bacterial community composition and its association with morel intake. **(A)** The relative abundance of top 10 taxa at genus level. Scatter plots showing the correlation between morel addition and the relative abundance of bacterial genera. **(B)** *Staphylococcus*, **(C)** *Bacteroides*, **(D)** *Lachnospiraceae NK4A136 group*, and **(E)** *Parabacteroides*. Solid line represents the regression line. The correlation coefficient (r) and *p*-value are reported on each plot.

At the genus level, the dominant taxa (relative abundance >1%) included *Dubosiella*, *Staphylococcus*, *Faecalibaculum*, *Allobaculum*, *Lachnospiraceae NK4A136 group*, *Bacteroides*, and *Parabacteroides* (Figure 2A), Among these genera, *Staphylococcus* was negatively correlated with morel intake (*R*^2^ < 0.21, *p* < 0.005) (Figure 2B). While *Bacteroides* (Figure 2C), *Lachnospiraceae, NK4A136 group* (Figure 2D), and *Parabacteroides* (Figure 2E) were identified as the positively correlated taxa with morel intake (*R*^2^ > 0.25, *p* < 0.005). Notably, at the species level, we found a positive correlation between the relative abundance of *P. distasonis* (Supplementary Figure S2A) and *B. thetaiotaomicron* (Supplementary Figure S2B) with morel intake (*R*^2^ > 0.3, *p* < 0.005), while the relative abundance of *S. lentus* showed a negative correlation (*R*^2^ = 0.159, *p* = 0.03) (Supplementary Figure S2C).

**Figure 3:**
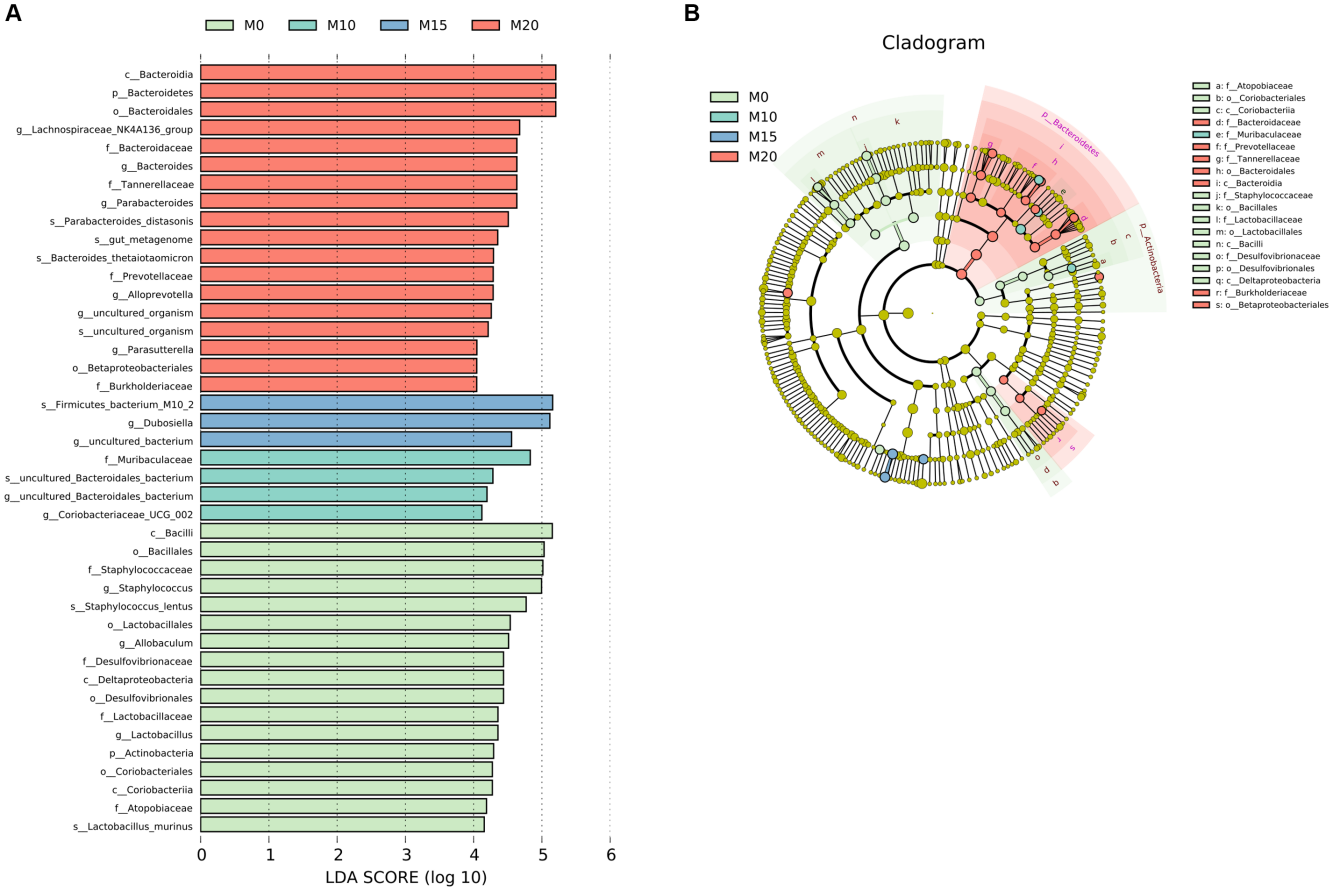
LEfSe analysis of microbial communities in the control and morel-intervention groups. **(A)** The logarithmic LDA (linear discriminant analysis) score of differentially abundant taxa between groups with cutoff of LDA score ≥ 4. **(B)** A cladogram illustrating the hierarchical relationships of taxonomic groups with different colors representing different taxonomic levels. The size of the circle indicates the abundance of each taxon in the group.

We performed a LEfSe analysis to identify the biomarker in the microbiota of control and morel-intervention groups (Figure 3). We observed 42 differentially abundant taxon with cutoff of LDA score > 4 (Figure 3A). Biomarkers enriched in M0, M10, M15, and M20 were 17, 3, 4, and 17, respectively. We found that the biomarkers enriched in the M0 group mainly belonged to family of *Atopobiaceae*, *Desulfovibrionaceae*, *Lactobacillaceae,* and *Staphylococcaceae*. The biomarkers enriched in M10 group were mainly from *Muribaculaceae*, while M15 was associated with *Dubosiella*. Biomarkers enriched in M20 were from the family of *Bacteroidaceae*, *Burkholderiaceae*, *Prevotellaceae,* and *Tannerellaceae* (Figure 3B).

### 3.3. Morel intervention increased the content of short-chain fatty acid in mice gut

**Figure 4:**
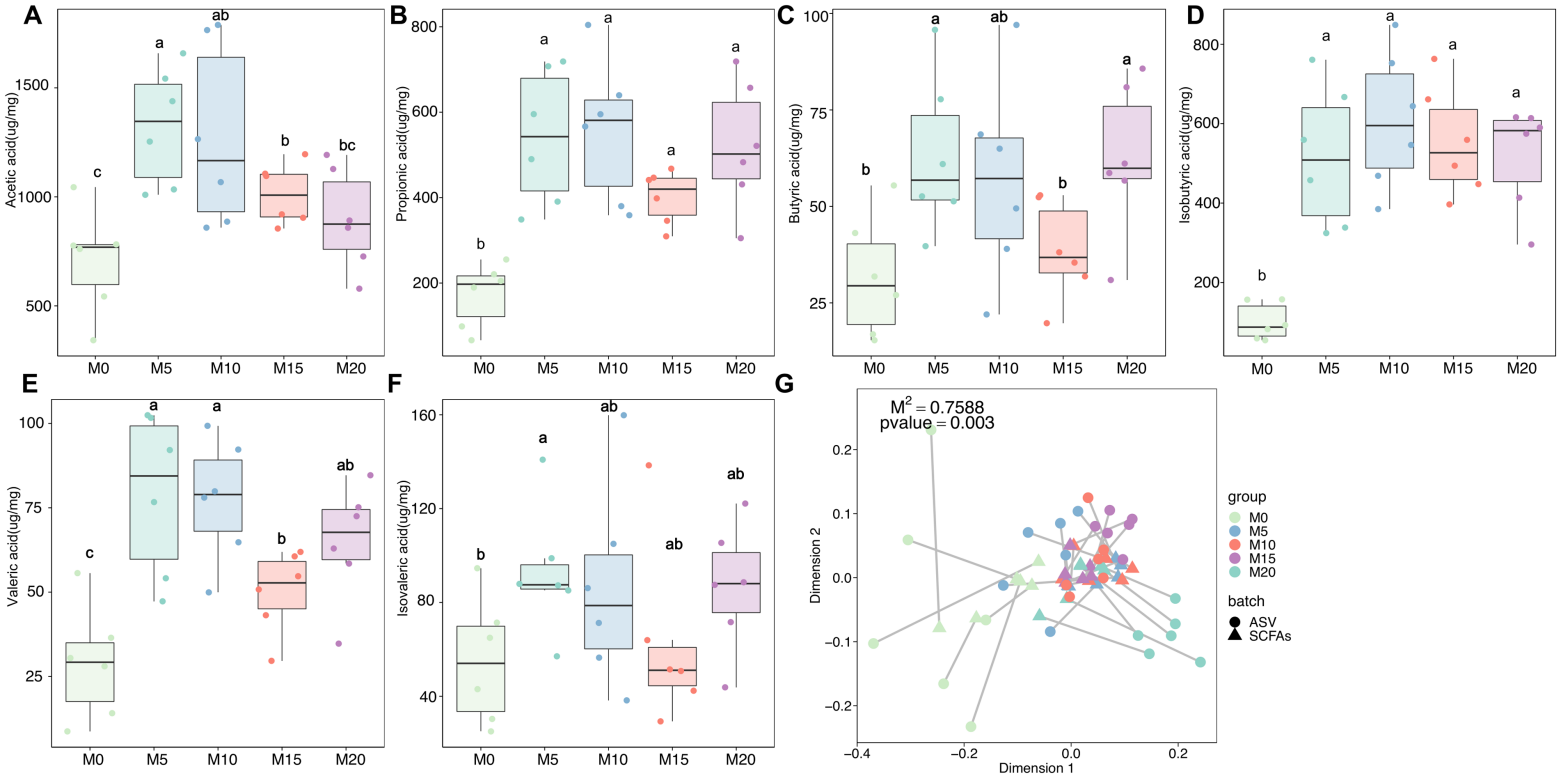
The variation in short-chain fatty acids (SCFAs) and their association with microbiota after morel intervention. Boxplot depicting the content **(A)** acetic acid, **(B)** propionic acid, **(C)** butyric acid, **(D)** isobutyric acid, **(E)** valeric acid, and **(F)** isovaleric acid in fecal samples from the control and morel-intervention groups. Statistical significance using one-way ANOVA is denoted by different letters (*p* < 0.05). **(G)** Procrustes analysis presenting the correlation between microbiota variation and SCFAs profile.

SCFAs are primary metabolites synthesized by the gut microbiota that have a significant impact on host health and disease. Therefore, we determined the content of six SCFAs, including acetate, butyrate, isobutyrate, valerate, isovalerate, and propionate in feces after morel intervention. In comparison to the control group (M0), we observed a significant upregulation of all SCFAs in the morel-intervention groups (Figure 4). Specifically, we noted a decrease in acetate content as the increase of morel intake (Figure 4A). However, there was an initial rise in butyrate, valerate, and isovalerate with increasing morel intake, followed by a decreasing trend then raised in M20 (Figures 4C,E,F). Additionally, propionate and isobutyrate content was higher in M10 than in the other morel-intervention groups (Figures 4B,D). We applied Procrustes analysis to test for gut microbiota variation and SCFAs across samples, which revealed a significant association between them (M^2^ = 0.759, *p* = 0.003, Figure 4G). To explore the potential SCFAs-producing bacteria, we conducted a correlation analysis between microbiota and SCFAs using MaAslin2. As is shown in Supplementary Figure S3, our analysis revealed a positive association between *Muribaculaceae* and propionate, as well as the moderate relationship with butyrate. We also identified *Jeotgalicoccus* (ASV_72) and *Gemella* (ASV_144) as the potential acetate-producing bacteria. Only one taxon, ASV_372 (*Lachnospiraceae*), was identified as potentially producing isobutyrate. *Odoribacter* (ASV_177), *Tyzzerella 3* (ASV_233), and *Ruminococcaceae UCG-014* (ASV_310) were correlated with valerate. Detailed information was shown in Supplementary Table S2.

### 3.4. Morel intervention changed the function of mice gut microbiota

**Figure 5:**
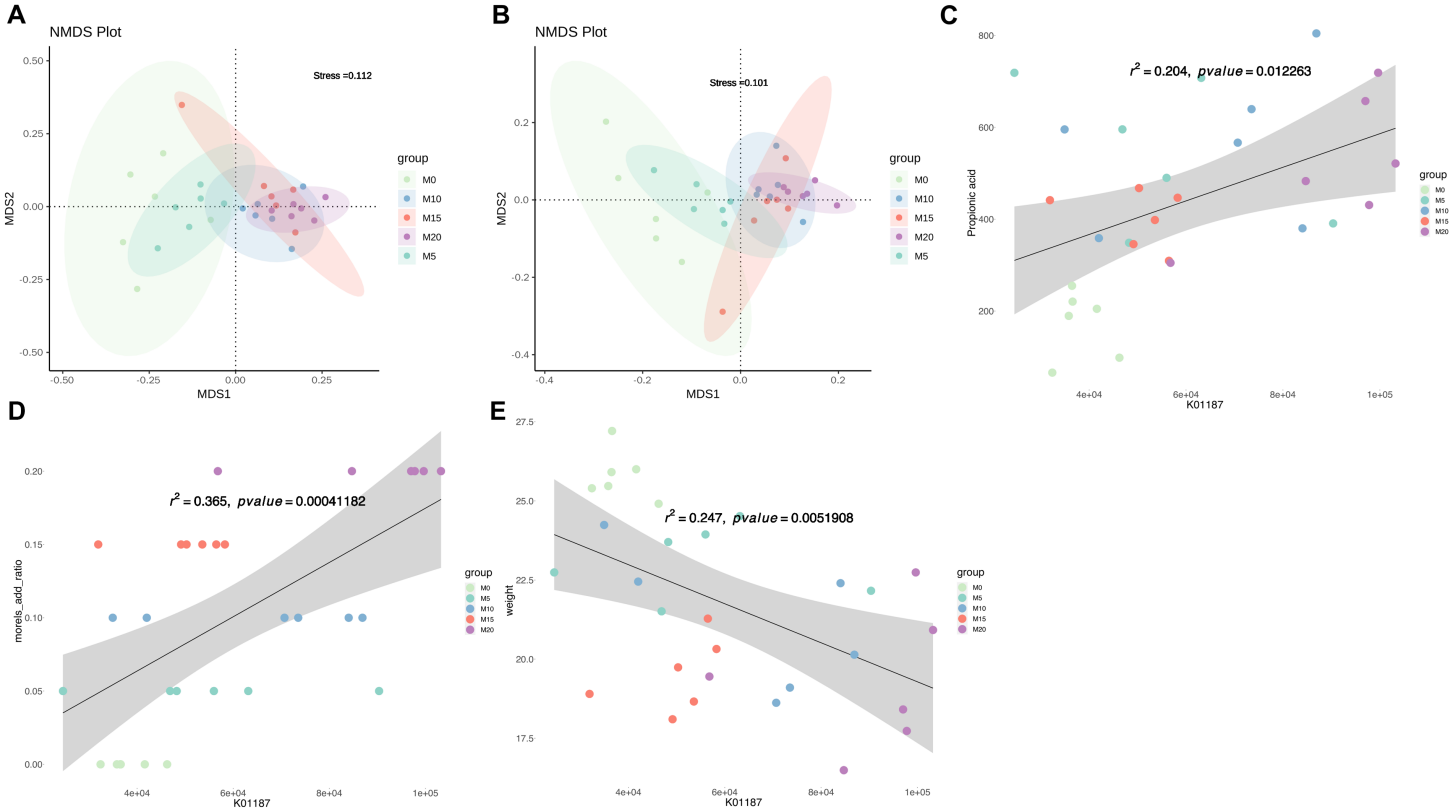
Predicted microbiota function by PICRUSt2. Non-metric multidimensional scaling (NMDS) plot based on **(A)** KO abundance and **(B)** COG abundance showing the functional differences in the microbiota between samples. Scatter plots showing the correlation between abundance of K01187 (alpha-glucosidase) and **(C)** propionic acid content, **(D)** morel intake, as well as **(E)** weight. Solid line represents the regression line. The correlation coefficient (*r*) and *p*-value are reported on each plot.

The function of mice gut microbiota was predicted using PICRUSt2 and NMDS analysis was performed to compare the microbial function profiles at both KO and COG levels among different groups. The results revealed distinct differences in the functional profiles, particularly between the control and intervention groups (Figures 5A,B). Further analysis using MaAslin2 identified significant associations between morel intervention and KEGG pathways (Supplementary Figure S4), such as ko00120 (primary bile acid biosynthesis) and ko00121 (secondary bile acid biosynthesis), as well as moderate associations with these pathways, such as ko00500 (starch and sucrose metabolism), ko00521 (streptomycin biosynthesis), and ko00520 (amino sugar and nucleotide sugar metabolism), among others. Moreover, the levels of certain SCFAs were found to be highly correlated with specific pathways. For instance, acetate was strongly correlated with ko03050 (Proteasome) and ko02060 (Phosphotransferase system), isobutyrate with ko00730 (Thiamine metabolism), and propionate with ko04974 (Protein digestion and absorption). These results provide insights into the potential roles of gut microbiota and their metabolic functions in response to dietary interventions in mice. LEfSe analysis based on KEGG orthologies (KOs) revealed that a total of 579 differentially functional catalogs were identified, among which 233, 3, 40, 50, and 263 function biomarkers enriched in M0, M5, M10, M15, and M20, respectively (Supplementary Table S3). We further revealed several representative enzymes assigned to specific KEGG pathways, including K05349 (beta-glucosidase), K01190 (beta-galactosidase), K12373 (hexosaminidase), K01991 (polysaccharide biosynthesis/export protein), K01187 (alpha-glucosidase), and K05989 (alpha-L-rhamnosidase) (Supplementary Table S3). Regression analysis demonstrated that these KO abundances were significantly correlated with morel intake, propionate or isobutyrate production and weight reduction (Supplementary Table S4). For instance, K01187 abundance was positively correlated with propionic acid content (*R*^2^ = 0.204, *p* = 0.01, Figure 5C), and morel intake (*R*^2^ = 0.365, *p* = 0.0004, Figure 5D), while a negative correlation was found with host’s weight (*R*^2^ = 0.247, *p* = 0.005, Figure 5E).

## 4. Discussion

Diet has a significant impact on the composition of the human gut microbiome. Dietary changes, both short-term and long-term, affect the ecology of the gut microbiome. A growing body of research has shown that dietary interventions are an effective strategy for regulating and improving gut microbiota, which plays a crucial role in preventing a range of chronic diseases and intestinal injuries (29, 30). It has been reported that edible mushrooms, especially those high in dietary fiber (31), can modulate the gut microbiome through prebiotic, anti-inflammatory, and immune system support effects (32). Studies also found that morels exhibit anti-inflammatory and free radical scavenging effects (33), as well as hepatoprotective activity (34). Although research has be conducted on other mushrooms and their extracts can have a health impact on the host by improving the gut microbiota, such as *Agaricus bisporus* (35), *Pleurotus eryngii* (36), *Auricularia auricular* (28), *Antrodia cinnamomea* (37), and *Ganoderma lucidum* (38), limited studies have reported on how morels modulate the gut microbial composition and promote health (39).

Significant changes in gut microbial community structure and composition were observed following morel intervention. The F/B ratio, which varies depending on multiple factors, is associated with obesity and is considered an important indicator of body health and a healthy gut (40, 41). Furthermore, increased feeding of morel resulted in a decrease in F/B ratio, which represents a balanced or healthful gut micro-ecosystem, potentially holding promise for preemptive dietary interventions targeting prevalent chronic conditions, particularly obesity (42, 43). This notion finds reinforcement in the noteworthy decrease in body weight observed among mice within the intervention groups. *Bacteroides*, which were elevated in the morel-intervention groups in our study, are known to have excellent polysaccharides utilization abilities that affect food metabolism (44). *Bacteroidetes* possess an abundance of glycoside hydrolases (GHs) and polysaccharide lyases (PLs), resulting in the degradation of a wide range of polysaccharides (27). Additionally, *Bacteroides*, *Lachnospiraceae NK4A136 group* and *Parabacteroides* increased following morels intervention (Figures 2C–E), especially that *B. thetaiotaomicron* was demonstrated to have the ability to lower total and inguinal fat mass and reduce plasma glutamate concentration through K01580 (glutamate decarboxylase) (45). K01580 has the highest abundance in 20% morel-intervention group than in other groups (Supplementary Table S3). *P. distasonis*, one of the 18 core members in human gut microbiota (46), had metabolic benefits by modulating the production of succinate and secondary bile acids to reduce weight and other metabolic disorders (47, 48). Moreover, this species has been shown to produce SCFAs and regulate immune systems (49, 50). Notably, *Lachnospiraceae NK4A136 group* as a potential probiotic may play an important role in gut homeostasis and host prevention to influenza infection (51). These findings supported our results that morel intake enriched *P. distasonis* (Supplementary Figure S2A; Figure 3), increased the SCFAs (Figure 4; Supplementary Figure S3; Supplementary Table S2), leading to the decrease in food intake (Supplementary Figure S1D) and weight loss (Supplementary Figure S1C). *Staphylococcus lentus* has recently been identified as a pathogen in the sinonasal cavities (52) and is also a commensal bacterium in the skin and gut of animals, associated with skin infections (53, 54), following morels intervention, there was a decrease in its levels (Supplementary Figure S2C).

Morels are rich in various types of dietary fiber, a complex carbohydrate that resists digestion and absorption in the small intestine and reaches the colon, where it is fermented by the gut microbiota (2). The dietary fiber content of the morels used in the study was higher than AIN-93G (Supplementary Table S5). SCFAs are the main products of fiber fermentation in the gut (55). Our study found that morel intake of significantly increased the production of SCFAs, especially propionic, butyric and acetic acids, butyric acid exerts a stronger anti-inflammatory effect and inhibits bacterial active transport, they also enhance the integrity of the intestinal barrier, provides energy to colon cells, and regulate apoptosis, which is important for the prevention of colon cancer and the maintenance of intestinal health (56–58). Propionate is the end product of metabolism, and increased colonic propionate is a potential target for appetite regulation, while fiber intake is an important mediator of appetite suppression (59). Recent reports suggest that acetate can modulate the complex balance of gut microbiota by regulating microbiota components (60).

The metabolism of Firmicutes and Bacteroidetes in the intestine produces mainly butyric, propionic and acetic acids, with Bacteroidetes reported to be the largest propionate producer in the intestinal gut microbe (42, 61). Instead of identifying the commonly found SCFAs-producers, such as *Lactobacillus* and *Bifidobacterium* (62), we found that SCFAs may be related to the other taxa. For example, *Muribaculaceae* showed a fiber degrading capacity (63), which might be related to the production of SCFAs (64). *Eubacterium rectale* and *Roseburia* spp. belonging to *Lachnospiraceae* are major SCFAs-producers in the human colon (58, 65). In addition, the *Lachnospiraceae NK4A136 group* produces SCFAs by fermentation of dietary polysaccharides and has been negatively associated with a variety of metabolic diseases and chronic inflammation (66, 67). *Jeotgalicoccus* had a positive correlation with acetate, propionate and butyrate after soy isoflavone feeding in mice (68). More studies have also found that other taxa, such as *Gemella* (69), *Odoribacter* (70), and *Tyzzerella* (71), have the ability to produce SCFAs. However, as the potential SCFAs-producing species were only identified by statistical analysis in this study, further validation by deep genomic analysis combined with wet-experiments is required due to the limited sample size and low-resolution species identification of amplicon sequencing.

Previous studies have discovered that the fecal bile acids enhanced after feeding mushroom polysaccharides to HFD-induced animal models (36, 63), which supports our finding that the primary and secondary bile acid biosynthesis increased after morel intervention in mice gut microbiota. Most bacteria depend on the phosphotransferase system (PTS) to efficiently import carbohydrates into cells for further utilization (72), and similar results were observed for starch and sucrose metabolism after dietary intervention (73, 74). Morels are also a high-protein Chinese medicine that stimulates the gut microbiota to secrete many digestive enzymes, such as β-glucosidase, β-galactosidase, α-glucosidase, and rhamnosidase, to breakdown the carbohydrates and proteins and produce bioactive ingredients easily absorbed by the gut (75).

## 5. Conclusion

**Figure 6:**
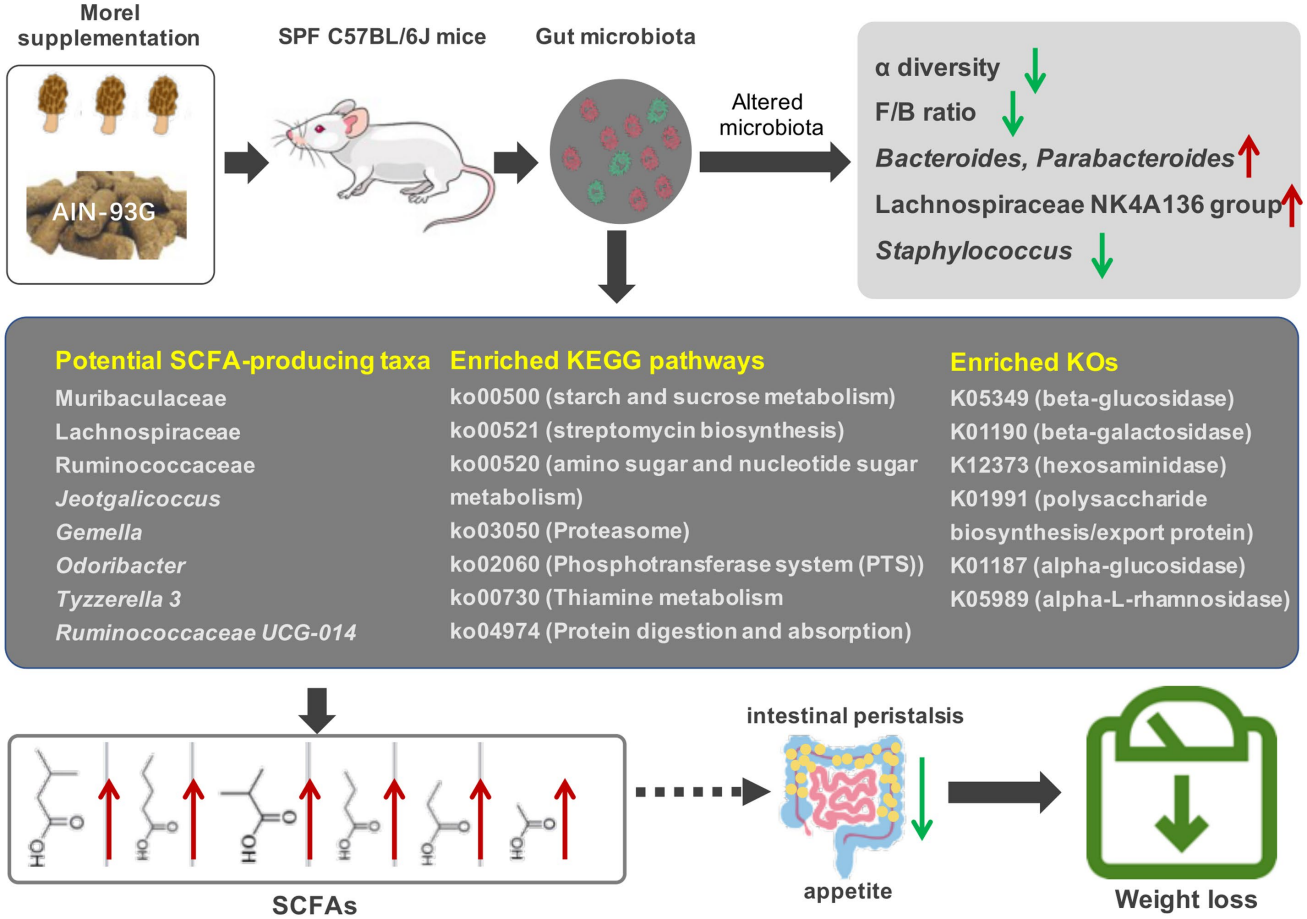
Effects of morel intervention on gut microbiota, short-chain fatty acid profile, and body weight.

Taken together, our results highlight the potential of morel (*Morchella* spp.) intervention to modulate the composition and function of gut microbiota, thereby influencing the production of short-chain fatty acids and host’s weight, and is possibly a dietary supplement that can improve intestinal health and prevent intestinal diseases (Figure 6). However, further research is necessary to (i) isolate and identify the active compounds that are responsible for the modulation effects; (ii) evaluate the impact of the host’s immune status in addition to their weight; and (iii) conduct in-depth investigations of the microbiota function using metagenomic sequencing. Overall, this study establishes a scientific foundation for inclusion of morels in the diet as a strategy for modulating gut microbiota and promoting weight loss.

## Data availability statement

The datasets presented in this study can be found in online repositories. The names of the repository/repositories and accession number(s) can be found in the article/Supplementary material.

## Ethics statement

The animal study was approved by Institutional Animal Care and Use Committee (IACUC) in China. The study was conducted in accordance with the local legislation and institutional requirements.

## Author contributions

LP: data curation, investigation, and writing – original draft. WL: investigation, and writing – review and editing. LL: investigation and methodology. XW: investigation. LJ: data curation. ZC: data curation, and writing – review and editing. QW: software. PW: methodology, and writing – review and editing. HX: investigation and data curation. All authors contributed to the article and approved the submitted version.

## Funding

This research was supported by the Central Guiding Local Science and Technology Development Special Fund Project (ZYYD2022C03), “Research and Demonstration of Key Technologies for Industrial Upgrading of Morel in Southern Xinjiang”; Xinjiang Institute of Technology-level scientific research project (ZZ202102), “Establishment of key cultivation techniques of morel in southern Xinjiang”; Graduate Student Research and Innovation Project of Xinjiang Production and Construction Corps (TDGRI202211), “Extraction of polysaccharides from morels based on low eutectic solvents and study of their beneficial properties”.

## Conflict of interest

ZC, QW, and PW were employed by Zhiran Biotechnology Co., Ltd.

The remaining authors declare that the research was conducted in the absence of any commercial or financial relationships that could be construed as a potential conflict of interest.

## Publisher’s note

All claims expressed in this article are solely those of the authors and do not necessarily represent those of their affiliated organizations, or those of the publisher, the editors and the reviewers. Any product that may be evaluated in this article, or claim that may be made by its manufacturer, is not guaranteed or endorsed by the publisher.

## Footnotes

^1^ https://github.com/picrust/picrust2
