# Supplementary material for: Morel (Morchella spp.) intake alters gut microbial community and short-chain fatty acid profiles in mice
Source: Front Nutr. 2023 Sep 22;10:1237237. doi: 10.3389/fnut.2023.1237237 (PMC10556497; doi:10.3389/fnut.2023.1237237)
Supplement: Supplementary file 1 [file Data_Sheet_1.docx]

**Supplementary Figures**

**Morel (*Morchella spp.*) Intake Alters Gut Microbial Community and Short-Chain Fatty Acid Profiles in Mice**

**
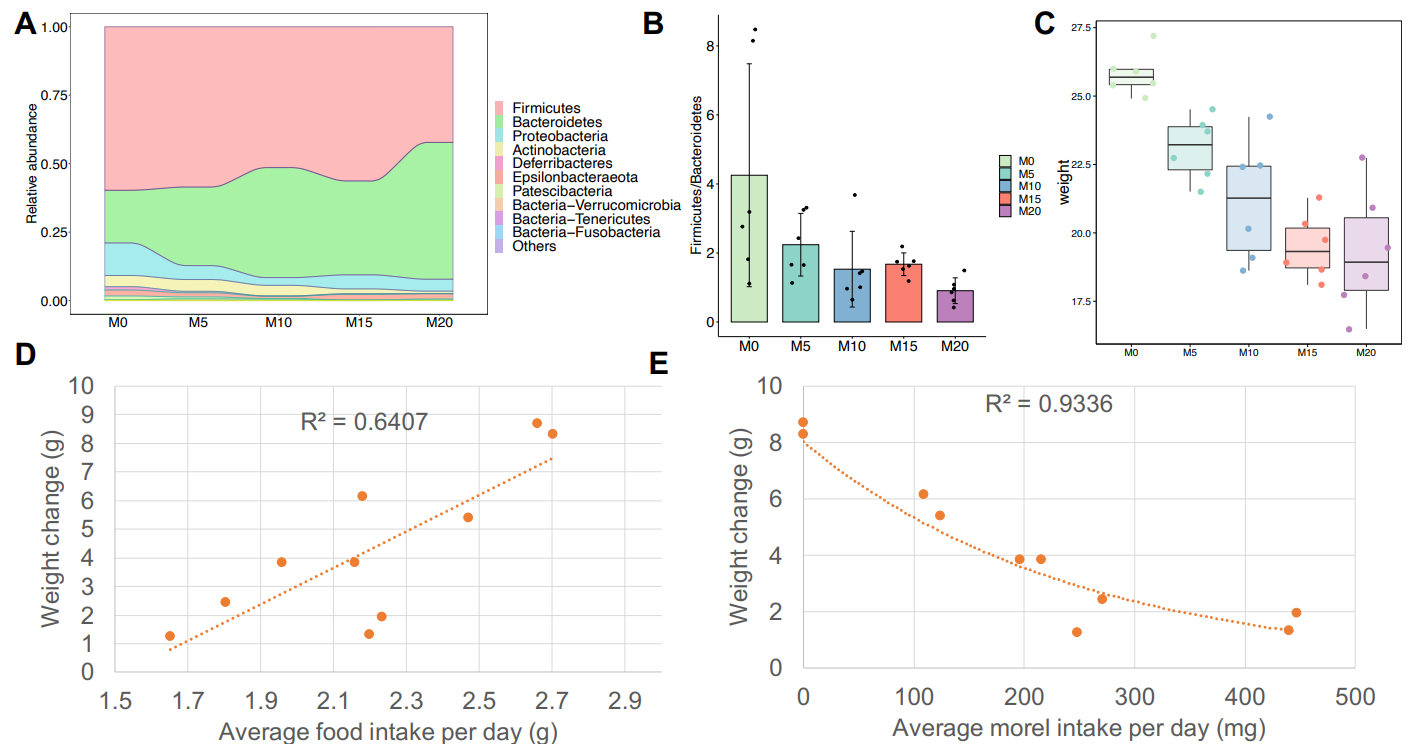
**

**Figure S1** The changes in relative abundance at phylum level (A), Firmicutes to Bacteroidetes ratio (B), and weight (C) at the end of experiment. The correlation scatter plot of weight change and food intake (D), morel intake (E).

**Figure S2** Scatter plots showing the correlation between morel addition and the relative abundance of bacterial genera. (A) *Parabacteroides* *distasonis*, (B) *Bacteroides thetaiotaomicron*, and (C) *Staphylococcus lentus*.

**Figure S3** The correlation analysis by MaAsLin2 between microbiota abundance and SCFA content. Color key denotes the coefficient value. “+” and “-” represent positive and negative correlation, respectively. Details are shown in Table S2.

**Figure S4** The correlation analysis by MaAsLin2 between KEGG pathway abundance predicted by PICRUSt2 and SCFA content. Color key denotes the coefficient value. “+” and “-” represent positive and negative correlation, respectively.
